# You Name It – How Memory and Delay Govern First Name Dynamics

**DOI:** 10.1371/journal.pone.0038790

**Published:** 2012-06-20

**Authors:** David A. Kessler, Yosi E. Maruvka, Jøergen Ouren, Nadav M. Shnerb

**Affiliations:** 1 Department of Physics, Bar-Ilan University, Ramat-Gan, Israel; 2 Department of Biostatistics and Computational Biology, Dana-Farber Cancer Institute, Boston, Massachusetts, United States of America; 3 Department of Biostatistics, Harvard School of Public Health, Boston, Massachusetts, United States of America; 4 Research Department, Statistics Norway, Oslo, Norway; Tel Aviv University, Israel

## Abstract

The adoption and abandonment of first names through time is a fascinating phenomenon that may shed light on social dynamics and the forces that determine cultural taste in general. Here we show that baby name dynamics is governed almost solely by deterministic forces, even though the emerging abundance statistics resembles the one obtained from a pure drift model. Exogenous events are shown to affect the name dynamics very rarely, and most of the year-to-year fluctuations around the deterministic trend may be attributed solely to demographic noise. We suggest that the rise and fall of a name reflect an “infection” process with delay and memory. The symmetry between adoption and abandonment speed emerges from our model without further assumptions.

## Introduction

Baby names have been considered, for a long time, as a particularly suitable model system for the study of cultural traits and social dynamics [1]–[4]. Most people pick a name for their baby from a pool of existing first names, and only a few give their baby a genuinely original name. Unlike many other products and styles, in general no commercial interest is involved in the process, and thus the name dynamics provides us with a relatively “clean” example of the rise and fall of a fashion, reflecting the underlying social network and the basic processes that take place on it.

The dynamics of first names varies tremendously among different societies and through time. The biblical corpus names thousands of people with unique names, and there are only a few repetitions of first names; in particular, out of the 45 kings of Judea and Israel mentioned in the Bible, there is not a single pair of identical names. In the Latin – Hellenistic world the differences between Greece and Roman cultural habits were emphasized by the historian Theodor Mommsen:

“… It seems as if the small and ever diminishing number and the meaningless character of the Italian, and particularly of the Roman, individual names, compared with the luxuriant and poetical fullness of those of the Greeks, were intended to illustrate the truth that it was characteristic of the one nation to reduce all to a level, of the other to promote the free development of personality. [5]”.

Later, in medieval Europe and in early modern times, the diversity of names was quite limited [6]: Henry VIII of England married six wives, among which three were Catherine, two Anne and one Jane. Since 1066, virtually all the kings of England were William, Henry, Edward, Charles, James or Richard, while the French kings were Lewis, Philip, and again Henry and Charles. The popularity of baby names was very robust until the end of the 18^th^ century [3] but since then names have become a matter of fashion, with a typical time evolution involving growth and decline.

In this paper we analyze the dynamics of first names in Norway. Relative to other nations in the western world, Norwegian society is fairly uniform. The residents of Norway are predominantly ethnic Norwegians who are of North Germanic/Nordic descent, and about 95% of them are either Lutheran Christians or came from Lutheran families.

The work presented here has two main goals. First we would like to demonstrate the deterministic nature of names dynamics. Second, we intend to suggest an underlying mechanism that explains the main features of this social spreading phenomenon.

We refer to “deterministic”, as opposed to “stochastic”, in two different ways. A few authors have suggested that exogenous events (“contamination” of a name associated with a notorious character or an increased popularity for a name associated with a movie star or a successful leader) play an important role in names dynamics. In population dynamics this effect is known as “environmental noise”, i.e., external random event affecting the growth rate of a population or the spread of an innovation. We will show that these external perturbations are extremely rare.

However, our system is affected by an intrinsic noise, known as demographic stochasticity. Two names with the same level of attractiveness will not score the very same number of babies at a given year, because of the stochastic nature of the decision making process by each pair of parents. This implies an intrinsic noise that scales with the square root of the expected number of babies. Hahn et al. [1], [2] have suggested that demographic noise is the *only* factor that shapes names dynamics, and that the process may be described adequately by a community drift model. We will track the name dynamics over time to show that demographic stochasticity plays only a minor role in the variations of first names popularity.

**Figure 1:**
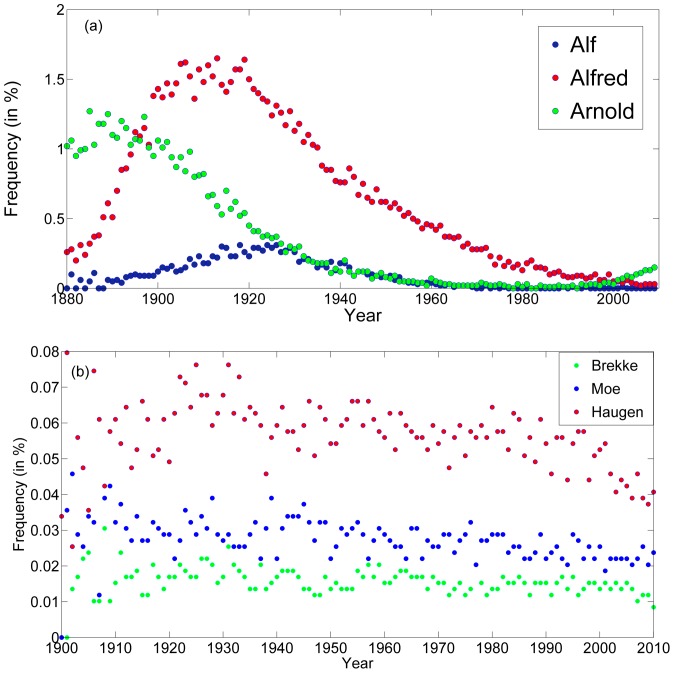
Frequency variations for three typical first names (a) and surnames (b) in Norway. The percentage (out of the total number of male birth during a certain year) of boys named Alf, Alfred and Arnold is plotted in the upper panel, and shows a pronounced pattern of rise and fall in popularity. Surnames like Brekke, Moe and Haugen show much weaker trends (if any, and demographic fluctuations are responsible for most of the variation.

Our second goal is to suggest a possible solution for a long standing puzzle: what causes the decline of a first name? Naively one can suggest that the popularity of a name is governed by a “word of mouth” dynamics: the parents select a name for their baby from a pool of possible names they encounter in their social circles. When a name becomes too popular, a negative response occurs, thus one should expect saturation at some popularity level. Instead, the measured popularity line shows (quite symmetric [4]) up and down trends. We suggest that the origin of these variations is the inherent delay in this system: the names one is exposed to do not reflect their current popularity among parents of new babies, but their popularity extending back over many years.

**Figure 2:**
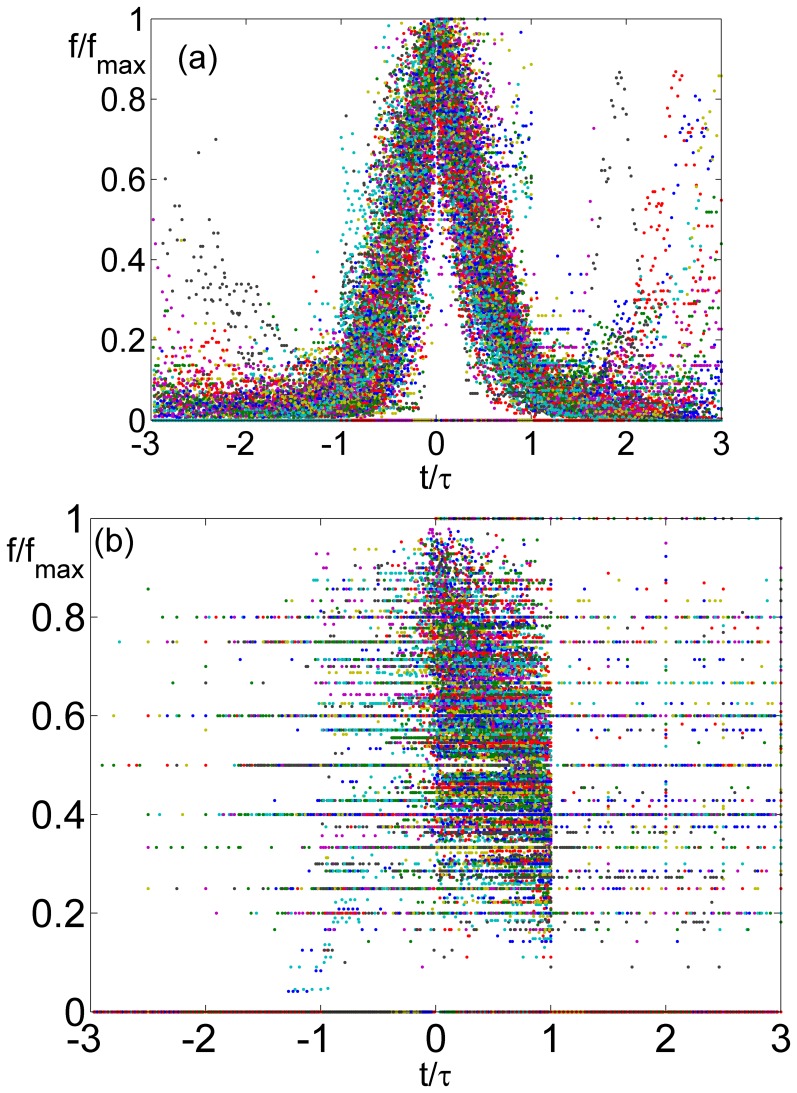
The frequency of all the boys’ first names is plotted in the right panel (a), for each name the frequency is divided by its maximal value and the timeseries were shifted such that the peak of all series appear at zero. Time for each name was normalized by the width of the peak τ, which is defined as the minimal number of years that takes for the name to fall to 10% of its peak value. The bell shape showing the process of rise and decline, and the “wings” showing reentrance of some names, are clearly seen. None of these is seen for surnames (b) when their abundance is plotted using the same procedure.

## Results

### Drift vs. Deterministic Dynamics

Figure 1 tells an interesting story. The upper panel shows the percentage of children born in Norway between 1880 and 2010 who carry the names Alf (black), Alfred (red) and Arnold (blue). In the lower panel, data for 1900–2010 is presented, also for Norway, but now what is presented is the number of newborns who carry the surnames Brekke, Moe and Haugen. The difference between these two graphs is striking.

**Figure 3:**
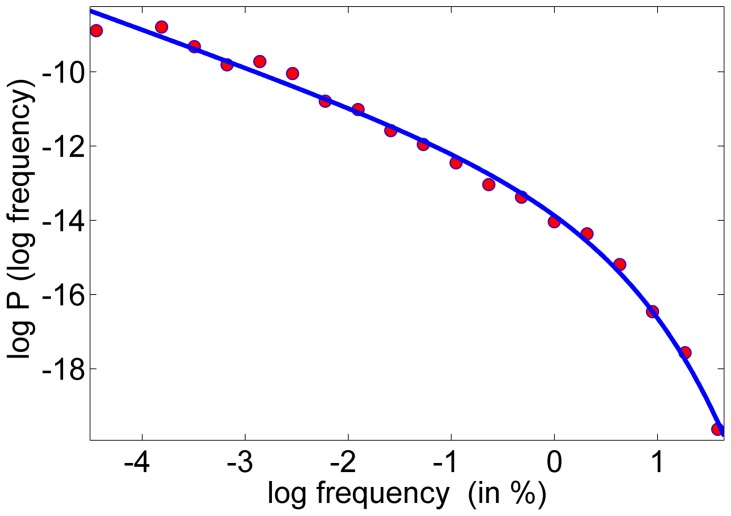
Name-abundance distribution curve for boys names. The number of names with abundance (frequency, in percent) *s* is plotted against *s* on a loglog scale (red circles), and shows a very good fit to the distribution suggested in Eq. 1 (blue line).

**Figure 4:**
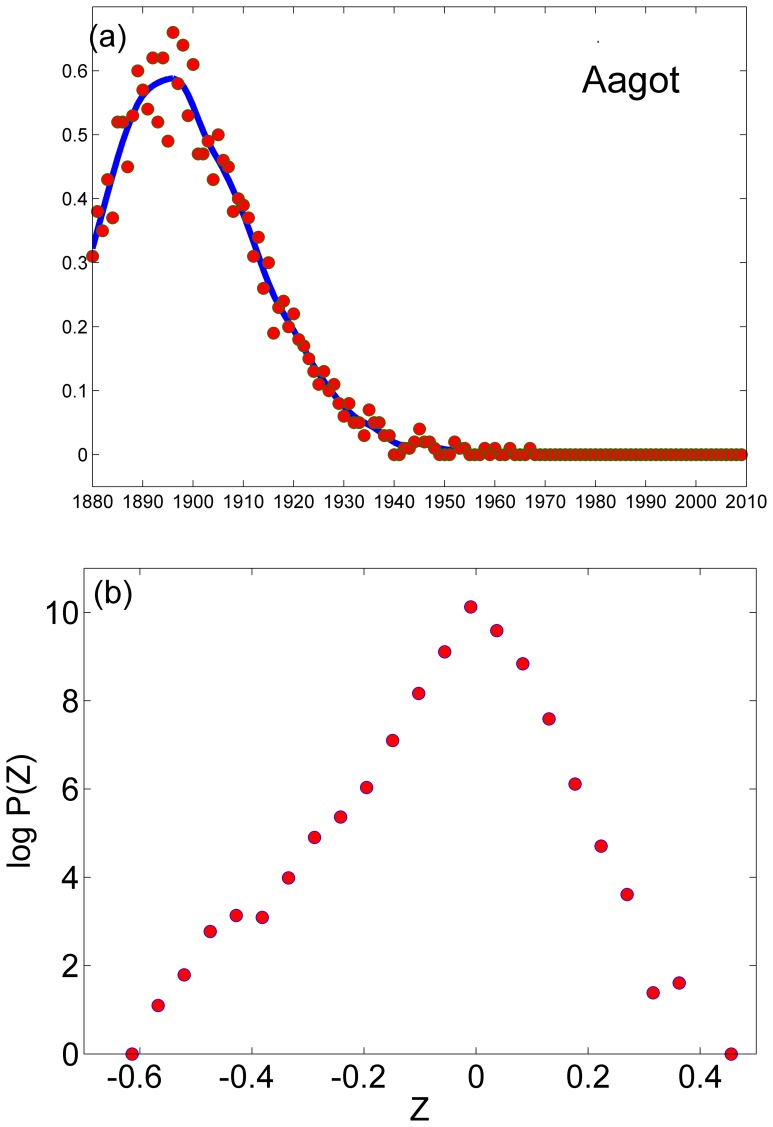
Quantifying stochasticity: the deterministic part of the dynamics was extracted from the timeseries associated with a given name using MATLAB’s smoothing algorithm, as shown in panel (a) for the first name in the girls’ list, Aagot. The distance from the real data (point) to the “prediction” of the deterministic dynamics (line), was then extracted for any year at each time series. After appropriate normalization for demographic noise [Eq. (2)] a histogram for the log of the deviations is shown in panel (b). The distribution of Z falls, more or less, exponentially with a width which is much smaller than unity.

Surname dynamics is fairly stochastic and may be described, quite well, by a community drift model [7]. In a traditional society where surnames are passed on from a father to his male descendants, the number of children with a certain surname reflects the frequency of this surname in the population, so if there are N_0_ Brekke’s in a population and the per-capita-per-year birth rate is γ, one should expect γ N_0_ newborns with this surname every year, up to demographic fluctuations that scale like the square root of this quantity. This is what one observes in Figure 1a: the fraction of each surname is more or less fixed, and the fluctuations are proportional to the square root of the absolute number. One can even notice the decrease in the relative amplitude of fluctuations over time; this phenomenon is related to the overall growth of the Norwegian population in time, such that the same percentage reflects more individuals.

**Figure 5:**
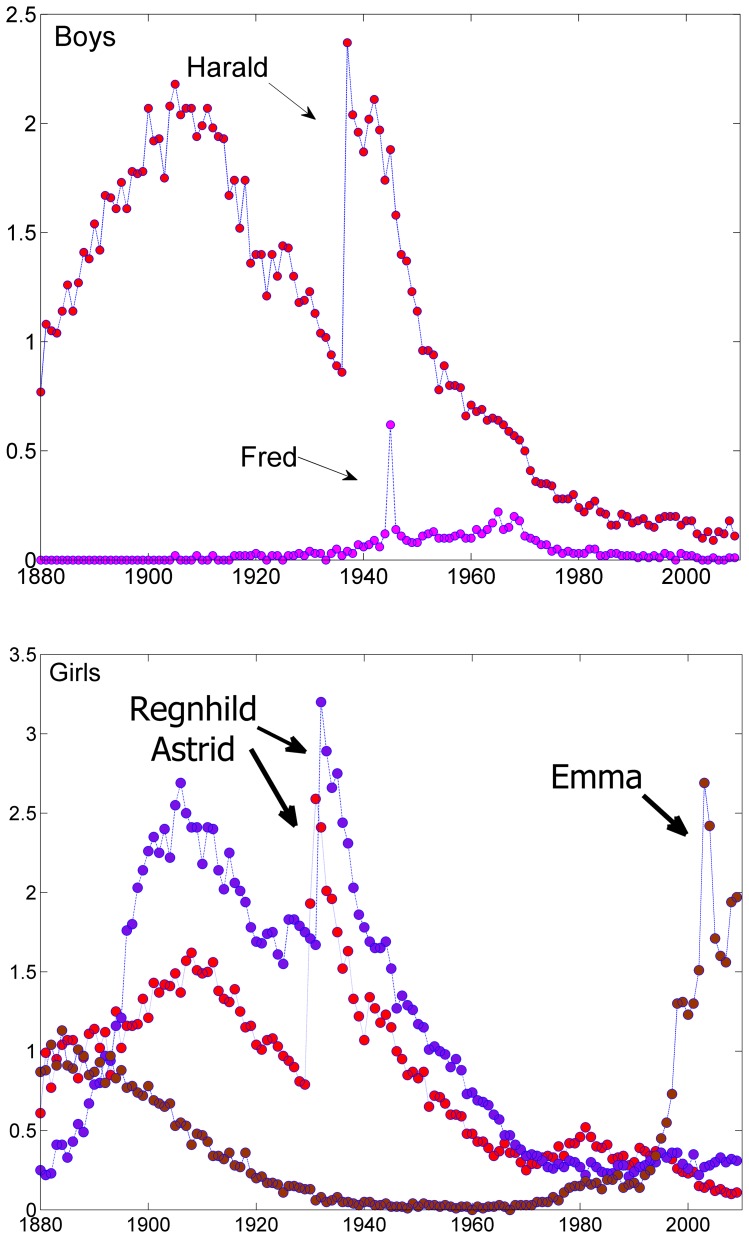
(Rare) examples of a pronounced exogenous factor that affects first name dynamics. Panel (a) shows three girls’ time series in which large and sudden changes are manifested. The two sharp rises in the frequency of Regnhild and Astrid in 1932 and 1933 are related to the birth of the princesses that carry these names at these years, correspondingly. The birth of prince Harald on 1937 explains the sudden popularity that this name gains in that year as shown in panel (b) for boys names. Fred (peace) became popular at the end of WWII, but we have no explanation for the dramatic rise of Emma on 2003.

**Figure 6:**
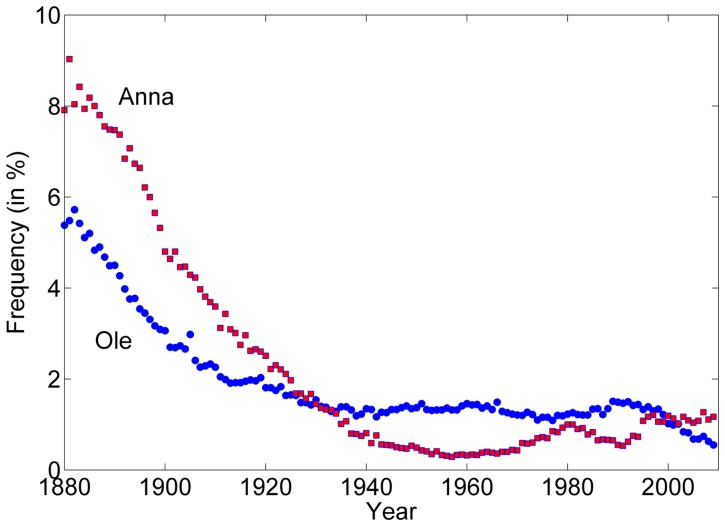
Overpopularity: The names Anna (for girl) and Ole (boy) were extremely popular at the end of the 19^th^ century, high above the level that prevents modern parents from using the name. The result was a sharp decline of these names frequency until they both reach the level of 1–2 percent, which is similar to the frequency of the most popular names nowadays.

What about first names? Here the dynamics is completely different. Although the 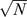 fluctuations are still there, the overall time evolution is clearly deterministic and cannot be attributed to mere stochasticity. Each first name has a “growth” phase, that reflects some sort of an “infection” process on the social network: the parents of a baby look around for “nice and original” names in their neighborhood – friends, coworkers, media stars. In this infection phase the dynamics may be described, quite nicely, by classical models of epidemics like the susceptible-infected-susceptible (SIS) or the susceptible-infected-susceptible (SIR) with a constant flow of susceptible individuals. After this phase the name became popular, then too popular, and its “attractiveness” decreases. This will be explained latter on as a classic **overshoot** phenomenon that occurs in many systems from physics to ecology, and in particular for logistic growth with delay. Finally, looking at the green (Arnold) dots, one can recognize the early onset of a new growth phase. This happens since the first name process has **memory**, as individuals name their babies after deceased family members.

**Figure 7:**
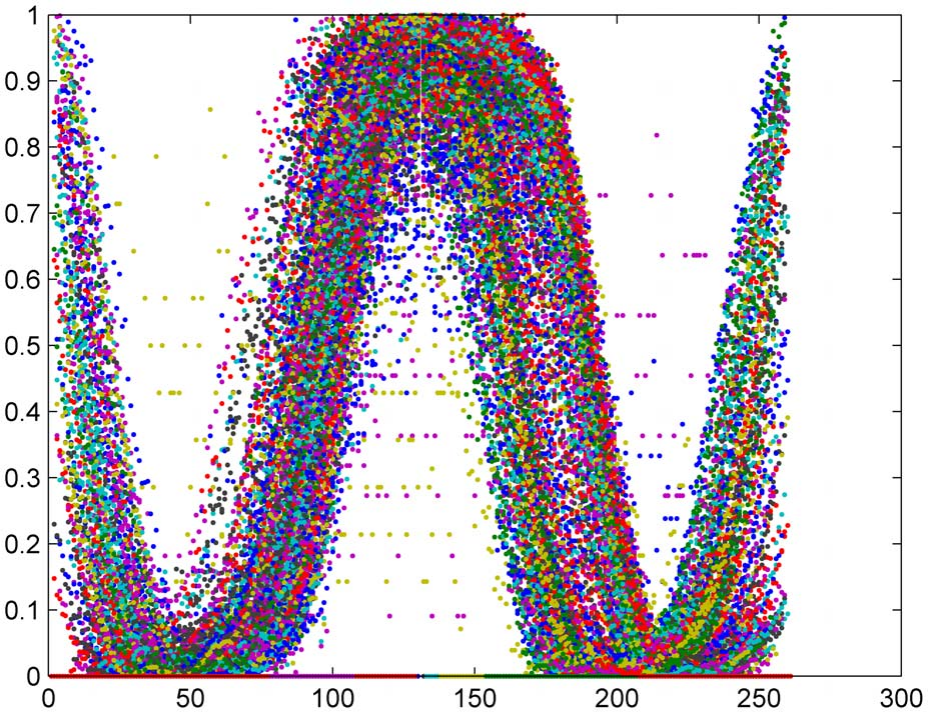
Simulated name dynamics for the toy model. We have simulated the dynamics of 500 “names” along 130 years, starting from random initial conditions, according to Eq. (3) and (4) with *a_d_ = a_m_* = 1, n = 20, n_1_ = 30 and τ = 80. We have shifted each of the time series such that the peak is always at zero and normalized the peak height to unity. While the result is not identical with the detailed features of real name dynamics emphasized in Fig 2a, it does show that both the bell shape and the “wings” appear when a simple logistic growth model includes both delay and memory.

Figure 2a emphasizes the characteristics of time evolution for male and female Norwegian babies’ names. We have plotted the number of babies that carry a certain name vs. time, where each time series was shifted such that the peaks of all the time series occur at t = 0. To compare the profile of names with different peak abundance and different duration of popularity, time was measured in units of τ, where τ is the number of years needed for this time series to reach 10% of its peak value (when both sides of the bell-shape appear in the data, we have taken the maximum of the two values). Peak abundance was normalized to unity. The regular bell-shape of the normalized data is pronounced; also one can recognize the “wings” associated with names that start to take off again after a period of decline. Note that, unlike avalanche processes that take place in many social and physical systems [8], the height of the peak does not scale with its width. The equivalent plot for surnames [Fig. 2b] shows no special features.

It is interesting to point out that, although the deterministic character of names variation is pronounced, the abundance statistics of these names is very similar to the celebrated Fisher log-series that appear naturally in system with a *pure* drift dynamics, like the community drift model of Hubbell [9], [10] and Kimura [11]. This feature, first discovered in [1], is depicted in Fig. 3, where the name abundance distribution, the probability of a name to appear in *m* percent of a given year’s cohort, (collected over all names along all years) is depicted. The fit to the Fisher log-series
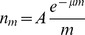
(1)is almost perfect with 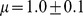.

To analyze the relative weight of stochasticity vs. deterministic dynamics, one would like to compare the fluctuations with the trend. To do so, the CSAPS Cubic smoothing spline algorithm of Matlab was implemented, as shown in Fig. 4a for the name Aagot, the first name in the girls’ list. The blue line is the result of the smoothing process with the smoothing parameter *p* = 0.05. Clearly, the fluctuations associated with the demographic noise, and perhaps also with the structure of the social network, are small in comparison with the trend. To examine this feature even further, we present in Fig. 4b a histogram of the relative fluctuations, i.e.,
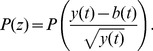
(2)


Here *y(t)* is the smoothed curve (e.g., the blue line in Fig. 4) and *b(t)* is the actual number of babies that carry this name at a given year. The distribution (presented here on a log normal scale) is a little bit skewed to the left, but it is *not* fat tailed. In fact, all the deviations associated with *z* values that are larger than 0.5 or so are associated with small absolute numbers (less than 5 babies) and most of them may be attributed to the fact that four is a threshold value (any name that was given to less than four babies at a certain year is reported as having zero number of birth for that year).

Indeed, for baby girls there are only three exceptions that one can find where large *z* values are associated with large number of babies. The relevant time series are shown in figure 5. Two of the jumps are attributed to the birth of the two princesses, Ragnhild and Astrid, in 1930 and 1932, correspondingly. The other pronounced jump is associated with the name Emma ca. 2003. For boys a very similar pattern appears, where the only large jumps are associated with Harald on 1937 (again, a birth of a prince) and with a very short-lived peak for the name Fred (which, in Norwegian, means “peace”) at the end of WWII in 1945. Note that these are only 5 exceptions, out of 1000 timeseries for 120 years! Significant exogenous events seems to be quite rare indeed.

## Analysis

### Names and the Delayed Growth Process

As explained above, names dynamics is, in principle, an infection process on the social network: parents select a name for their newborn baby from the set of names they were exposed to. Some people like rare names, other like more popular ones, but nobody would adopt a too popular name. In fact, during the last 200 years the standard for overpopularity have changed [3] [see the data for UK middle ages in [6]], and this tendency manifests itself also in Fig. 6. Not only have the most popular names at the end of the 19^th^ century, Anna and Ole, decreased in popularity almost monotonically ever since, no other name has risen to their level. In fact, the most frequent names today account for 1.5 percent of the boys and 2.5 percent of the girls, not even close to the 10% scored by Anna and 7% scored by Ole 120y ago.

But what leads to the inherent oscillations in the frequency of first name? Unlike many other fashions and products and as opposed to the predictions of epidemic models like those discussed above, names do not reach a fixed popularity level but show what looks like a regular up and down variations. We believe that the answer has to do with two effects: delay and memory. The delay leads to overshoots that end up in decline, while the memory leads to reoccurrence at long times and yields the oscillations.

There is a simple reason for the appearance of a delay: parent are not exposed to the frequency of names in the next hospital nursery, but to their abundance 5–40 years ago, as reflected in the names of their friends and their kids. Accordingly, people underestimate the popularity of any given name, and continue to use it even after it is already overexploited. It is well known that such a delay, when superimposed on a mechanism that supports growth with saturation (e.g., logistic growth) yields variations in time [12].

The other factor that leads to the recovery of names in the long run is the habit, in some cultures, to name a baby after a deceased relative. This leads to an inherent “memory” in the system, as names that were popular many years ago tend to regain popularity nowadays.

Although we cannot currently recover all the fine features of the dynamics from a model, we can try, at least, to demonstrate the feasibility of our hypothesis by showing that the main features of the dynamics do appear in a simulation of the most simple model that admits delay and memory. A fundamental model for growth and saturation of self-reproducing objects, from living animals to products and rumors, is the logistic process mentioned above. In this process an initial exponential growth is followed by saturation when the growth levels off. As will be shown below, when the process includes also memory and delay it yields a very nice cartoon of the observed names variations in time.

We have implemented a simple simulation to model all these aspects of name dynamics. Our toy model have a fixed population of *N* = 1000000 individuals, where the exposure of a name is given by
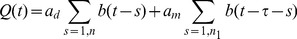
(3)where *b* is the number of babies with this name born at a certain year. The first term reflects the delay mechanism: if n = 20, for example, the parents’ exposure to a name is proportional to the number of babies with this name born in the last 20 years, *a_d_* is a constant scale factor. The next term describes the memory effect, as it accounts for those that carry the name and were born between τ and τ+n_1_ years ago, with *a_m_* another constant. Once *Q* is given, the number of births in a given year is taken from a Poisson distribution with average µ, where



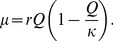
(4)This is a standard logistic growth term, where r is the linear growth rate. The last factor accounts for the effects of overexposure, where overuse leads to people devaluing a name. The constant κ sets the scale of exposure where this effect sets in. In the absence of memory, the name would saturate at a level κ. κ was chosen at random from an exponential distribution with mean N/10 where N is the size of the population; this fits more or less the statistics of peak values for first names. When 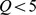, 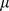 was taken to be 5 to account for the fact that the Norwegian data report values only for names with 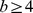. Initial conditions are random Poisson numbers with an average 1 for each year.

Simulating this system (with *a_d_ = a_m_* = 1, n = 20, n_1_ = 30 and τ = 80), and rearranging the results for the dynamics of 500 different names using the procedure we have implemented in Figure 2, we have obtained Figure 7. Although the details differ from the real data, and in particular the peak is more flat here, the general features of name dynamics are clearly seen. Given our limited ability to extract the various parameters from the data, and the use of the logistic term as a crude approximation for an unknown growth process with saturation, we consider these results as sufficient for a proof of principle.

We would like to stress that the symmetry between adoption and abandonment speed, demonstrated in Figure 2a, emerges naturally from our model dynamics without further assumption. There is no need to assume that parents actually remember the speed in which a given name caught in the past (many times this happened 30 years before) and respond to it today. The symmetry has to be attributed to the structure of the memory kernel and the exposure-attractiveness relations manifested in Eq. (3) and (4). It may be interesting to figure out the general conditions under which this and alternative models for the diffusion of an innovation or other social spreading phenomena [13], satisfy this kind of time reversal symmetry.

## Discussion

First names, like many other social memes, share a few common properties with ecological communities: many “species”, each has its own fitness and its own niche, are competing on a limited amount of resources. An intrinsic delay may appear also in population dynamics when the generation time is relatively large, and the Janzen–Connell [14], [15] hypothesis actually suggests that species diversity is maintained by mechanisms that limit the local density of a species, since an overcrowded patch attracts species-specific pathogens or predators. It will be interesting to find out if there are similarities between the names dynamics considered here and species abundance variations in real communities.

A major dispute among ecologists has to do with the factors that shape community structure. While traditional approaches attribute the composition of a community to deterministic factors like selection, fitness and competition, some modern theories [9] suggest the stochastic drift as the main cause of the observed patterns. In previous works we and others have considered surname dynamics [7], [16] and show that it satisfies quite nicely the main assumptions of a community drift model. Here we have shown that first names variations are actually governed by deterministic factors, but there is one important ingredient that we have borrowed from the “neutral” theories: the assumption that different names evolve *independently*, i.e., that the effects of other “species” on the popularity of a given name is negligible. This allows us to suggest a model where the current and the past frequency of a name, together with stochasticity, determine its future popularity.

Such an assumption is by no means trivial. Since names compete with each other for a common and limited “resource” – the number of babies born at a given year – one may suggest that their relative frequencies are strongly correlated. This must be true if there are only a few possible names, but it seems that, when many names compete with each other and there is an intrinsic limitation on the popularity of a single name, one may implement a “mean field” approach where the overall pressure from all other names is considered as a fixed background that does not evolve during the lifespan of name popularity. As we have seen, this approach actually provides very nice results, and perhaps one may apply it to other complex systems where many species or agents interact but (for some reason) none may take over a substantial part of the population.
